# Application of a multidimensional computerized adaptive test for a Clinical Dementia Rating Scale through computer-aided techniques

**DOI:** 10.1186/s12991-019-0228-4

**Published:** 2019-05-17

**Authors:** Yi-Lien Lee, Kao-Chang Lin, Tsair-Wei Chien

**Affiliations:** 1 Department of Medical Affairs, Chi-Mei Medical Center, No. 901, Chung Hwa Road, Yung Kung Dist., Tainan, 710 Taiwan; 2 Institute of Information Management, National Chung Cheng University, Chiayi, Taiwan; 3 Department of Neurology and Holistic Care Unit, Chi-Mei Medical Center, Tainan, Taiwan; 4 Department of Medical Research, Chi-Mei Medical Center, 901 Chung Hwa Road, Yung Kung Dist, Tainan, 710 Taiwan; 5 Department of Hospital and Health Care Administration, Chia-Nan University of Pharmacy and Science, Tainan, Taiwan

**Keywords:** Dementia, Clinical Dementia Rating (CDR), Computer adaptive testing, Multidimensional, Cut-off point

## Abstract

**Background:**

With the increasingly rapid growth of the elderly population, individuals aged 65 years and above now compose 14% of Taiwanese citizens, thereby making Taiwanese society an aged society. A leading factor that affects the elderly population is dementia. A method of precisely and efficiently examining patients with dementia through multidimensional computer adaptive testing (MCAT) to accurately determine the patients’ stage of dementia needs to be developed. This study aimed to develop online MCAT that family members can use on their own computers, tablets, or smartphones to predict the extent of dementia for patients responding to the Clinical Dementia Rating (CDR) instrument.

**Methods:**

The CDR was applied to 366 outpatients in a hospital in Taiwan. MCAT was employed with parameters for items across eight dimensions, and responses were simulated to compare the efficiency and precision between MCAT and non-adaptive testing (NAT). The number of items saved and the estimated person measures was compared between the results of MCAT and NAT, respectively.

**Results:**

MCAT yielded substantially more precise measurements and was considerably more efficient than NAT. MCAT achieved 20.19% (= [53 − 42.3]/53) saving in item length when the measurement differences were less than 5%. Pearson correlation coefficients were highly consistent among the eight domains. The cut-off points for the overall measures were − 1.4, − 0.4, 0.4, and 1.4 logits, which was equivalent to 20% for each portion in percentile scores. Substantially fewer items were answered through MCAT than through NAT without compromising the precision of MCAT.

**Conclusions:**

Developing a website that family members can use on their own computers, tablets, and smartphones to help them perform online screening and prediction of dementia in older adults is useful and manageable.

**Electronic supplementary material:**

The online version of this article (10.1186/s12991-019-0228-4) contains supplementary material, which is available to authorized users.

## Background

Dementia is a serious disorder that is appearing with increasing frequency in adults aged older than 60 years [1] and is a costly disease in terms of personal suffering and economic loss [1, 2]. Dementia is characterized by the loss of function in multiple cognitive domains [3] and directly influences patients’ daily living and social activities [4].

One of the most frequently used tools for examining the extent of dementia is the Clinical Dementia Rating (CDR) [5]. CDR is a problem-oriented questionnaire that is completed by the patient and their family members to examine the extent of dementia of the patient [6].

### Problems and requirements of the CDR in Taiwan

The CDR comprises two questionnaires, with 49 items for family members and 25 items for patients. Considerable time is needed to answer all items on the eight subscale domains, namely, memory, orientation, judgment, community affairs, home hobbies, personal care, personality and behavioral problems, and language. The assessment process is tedious, time-consuming, and subjective for the hospital technicians performing the CDR. In addition, the CDR assessment result is required for various uses, such as payments for medicines to managing Alzheimer’s disease (such medicines are regulated by the Taiwan government insurance institute) and/or for the employment of foreign caregivers who are hired by patients’ families. The demand for CDR certification is, thus, increasing in Taiwan, where the number of patients with dementia has reached 124,263, accounting for 0.54% of Taiwan’s approximately 23 million residents in 2017. Therefore, methods that use computerized assessment, particularly computerized adaptive testing (CAT) [7], to reduce the burden of technicians in CDR administration are urgently needed.

### Requirement for multidimensional computerized adaptive testing

CAT requires fewer items to be answered than the traditional pen-and-paper approaches (an efficiency gain of 32%), thereby suggesting a reduced burden for respondents [7, 8]. However, online CAT-based assessment is administered on a one-dimensional scale only rather than with multidimensional subscales (e.g., the eight-domain CDR) that capture the complexity of multidimensionality and CAT. This approach is called multidimensional CAT (MCAT) [9, 10]. Till now, only eight papers were found based on the keywords (multidimensional computerized adaptive testing [Title]) searched in Medline. Thus, we used MCAT to simultaneously estimate person measures for an inventory that consists of multiple subscales [11].

## Objectives

First, we compared MCAT with non-adaptive testing (NAT) based on efficiency and precision. Second, we determined a set of cut-off points that can be used for computing the extent of dementia for patients through MCAT. Third, we developed an online MCAT module for family members to measure the extent of dementia.

## Methods

### Study participants

The CDR scale was applied to 366 outpatients with dementia diagnoses at a 1236-bed medical center in Taiwan from June to September 2013. All CDR data were collected, including questionnaires from both patients and family members [12]. For simplicity, we merely adopted data from family members responding to the eight-domain, 53-item CDR designed by Ref. [12]. Consistent with the standard, the extent of dementia is categorized into five degrees: healthy (CDR 0), very mild (CDR 0.5), mild (CDR 1), moderate (CDR 2), or severe (CDR 3) [13, 14]. In this study, we applied the Rasch model [15] to the multidimensional random coefficients’ multinomial logit model [16]. The score was measured as a unit of logit (i.e., log odds), that is, the odd is equal to 1 (= exp[0]) when the logit is zero. By contrast, the logit equals zero (= log [1]) when the odd is 1.0. Higher logit scores indicate more severe cases of dementia for individuals.

This study was approved and monitored by the Research Ethics Review Board of the Chi-Mei Medical Center. Demographic data were anonymously collected.

### CDR subscales

The CDR was developed by the Memory and Aging Project at the Washington University School of Medicine in 1979 for the evaluation and severity staging of dementia [5, 17, 18]. It consists of 49 and 25 items that are answered by family members and patients, respectively. We adopted the two tools with 53 and 41 items used for MCAT from Ref. [12], which are answered by family members and patients, respectively, and applied Rasch ConQuest software [19] to calibrate item parameters (i.e., item threshold difficulties) in a logit unit. The overall item average difficulty was set at zero and used to estimate person measures. A higher response to an item indicates easier for a person to respond. Otherwise, a harder item implies a lower person measure to estimate.

### Simulation data

**Fig. 1:**
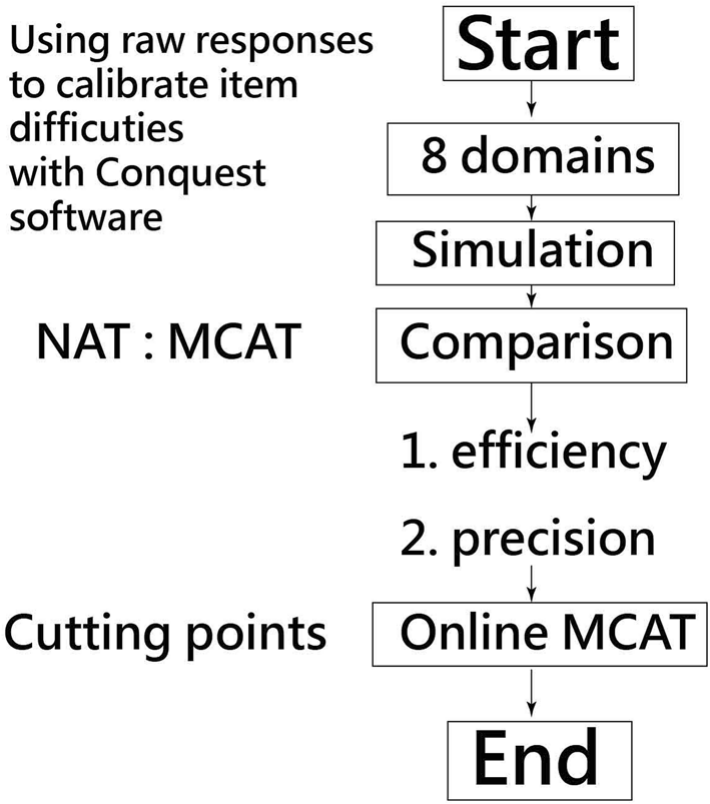
Flowchart of this study

The traditionally obtained original raw scores from family member responses were used for NAT to compare with the results of MCAT. We, thus, simulated responses to compare the efficiencies and precisions of MCAT and NAT based on the original responses. The number of items saved and the estimated person measures was compared between the results of MCAT and NAT, respectively (Fig. 1 and Additional file 1).

### Using Rasch analysis to estimate person measures

In classical test theory, on the basis of the assumption that all item difficulties are equal, raw summation scores (or linearly transformed scores such as the T-score) are often assigned as estimations of the examinee’s ability. However, this assumption is not true in the real world. Thus, we applied item response theory (IRT) based on Rasch probability model [15] to calibrate item difficulties and estimate person measures [20].

### Using MCAT to estimate person domain scores

The development of IRT in conjunction with advances in computer technology has made MCAT feasible and applicable [10]. Thus, we can consider using MCAT to simultaneously estimate the person measures for an inventory that consists of multiple subscales.

Traditionally, we perform CAT for each subscale separately. In general, MCAT is more efficient than separate unidimensional CAT in terms of reducing test length [11, 20], because MCAT assesses all subscales jointly and simultaneously. Detailed information regarding the simulation approach and MCAT estimation process is provided in Additional file 2.

### Cut-off points for CDR

Many scales have only one cut-off point (e.g., the cut-off at 12 on a depression scale). Straus et al. [21] proposed multiple cut-off points for a scale that can provide technicians with more information for decision-making [22].

Studies have revealed that as a scale’s reliability (i.e., Cronbach’s α) increases, the number of person strata can be confidently distinguished and increased [23–25]. Person measures with reliabilities of 0.67, 0.80, 0.90, 0.94, 0.96, and 0.97 will tend to vary, respectively, toward two, three, four, five, six, and seven strata [26].

For a more conservative approach to computing the number of strata, the scale reliability can refer to the Rasch person separation reliability. The Rasch threshold difficulty guideline [27] also recommends an appropriate distance between two thresholds ranging from 1.4 to 5.0 logits. In addition, an equal sample size in each stratum, as suggested by Maslach et al. [28], was applied to determine cut-off points.

Accordingly, a threshold at zero logits is logically suggested for two strata, −0.7 and 0.7 (= 1.4-logit difference with probabilities of 0.33 and 0.67 = 1 − exp [− 0.7]/{1 + exp [− 0.7]}) for three strata [7], −1.1, 0.0, and 1.1 (= 1.1-logit difference with probabilities of 0.25, 0.50, and 0.75 = 1 − exp [− 1.1]/{1 + exp [− 1.1}]) for four strata, and − 1.4, − 0.4, 0.4, and 1.4 (= 1.0-logit difference with probabilities of 0.20, 0.40, 0.60, and 0.80 (= 1 − [− 1.4]/{1 + exp [− 1.4]}) for five strata [7].

### Online MCAT for smartphones

All MCAT items (called an item pool) in this study can be applied to the Rasch partial credit model (i.e., each item has a different number of categories to be answered from other items). An online MCAT was particularly designed for patient family members to enable them to assess the extent of patient dementia. The 53 items with their threshold difficulties (calibrated using ConQuest software [19]; Additional file 3) and their responsive audios and pictures were uploaded to the website. The rules of the first and the next selected MCAT item, as well as the termination criteria are similar to those of our previous study [7].

### Statistical tools and data analyses

The ConQuest analysis displays correlation coefficients and covariance across subscales shown in Additional file 3. Independent *t* tests were used to compare the ratios of the different paired person measures on the mean scores of NAT and MCAT using the formula (= (*A1 − A2*)/√(*SE1*)2 + (*SE2*)2))) [29], where *A1* and *A2* are the mean person measures for NAT and MCAT, respectively, and *SE1* and *SE2* denote the standard errors for NAT and MCAT, respectively. If the difference is less than 5% (i.e., < 18 = 366 × 5%), then the inference that the two approaches (i.e., NAT and MCAT) display no difference can be made with 95% confidence. The difference in item length was compared between NAT and MCAT by using a paired *t* test. SPSS 21.0 for Windows (SPSS Inc., Chicago, IL, United States) and ConQuest were used to calculate Cronbach’s α.

## Results

### Patients’ demographic and clinical data

A total of 366 outpatients (188 males [51.37%] and 178 females [48.63%]) who were diagnosed with dementia were enrolled in this study, as shown in Table 1. The top three classifications of relatives came from the patients’ sons (70; 19.13%), spouses (66, 18.03%), and daughters (63; 18.03%), as shown in Table 2.

**Table 1:** Patients’ demographic and clinical data

Variable	Male	%	Female	%	Total
*Age*					
< 60	18	81.82	4	18.18	22
< 70	23	65.71	12	34.29	35
< 80	63	45.00	77	55.00	140
< 90	65	46.43	75	53.57	140
≥ 90	10	34.48	19	65.52	29
*CDR score(extent of dementia)*					
Healthy (CDR 0)	5	62.50	3	37.50	8
Very mild (CDR 0.5)	18	72.00	7	28.00	25
Mild (CDR 1)	56	56.57	43	43.43	99
Moderate (CDR 2)	59	42.75	79	57.25	138
Severe (CDR 3)	41	42.71	55	57.29	96
*Education*					
Elementary or under	53	27.04	143	72.96	196
Junior high	76	69.09	34	30.91	110
Senior high	34	85.00	6	15.00	40
College	3	50.00	3	50.00	6
University or above	13	92.86	1	7.14	14
*Occupation*					
Agriculture	36	38.71	57	61.29	93
Home service	28	33.33	56	66.67	84
Industry	26	59.09	18	40.91	44
Public official	31	43.06	41	56.94	72
Army	14	100.00			14
Teacher	1	50.00	1	50.00	2
Commerce	21	84.00	4	16.00	25
Others	31	96.88	1	3.13	32
Total	188	51.37	178	48.63	366

**Table 2:** Relatives’ information to patients

	*n*	%
Sons	70	19.13
Spouses	66	18.03
Daughters	63	17.21
Father-sons	59	16.12
Father-daughters	45	12.30
Daughters-in-law	39	10.66
Grand sons/daughers	11	3.01
Sons-in-law	6	1.64
Brothers/sisters	4	1.09
Nephews/nieces	3	0.82
Total	366	100.00

### MCAT for person and item analyses

**Fig. 2:**
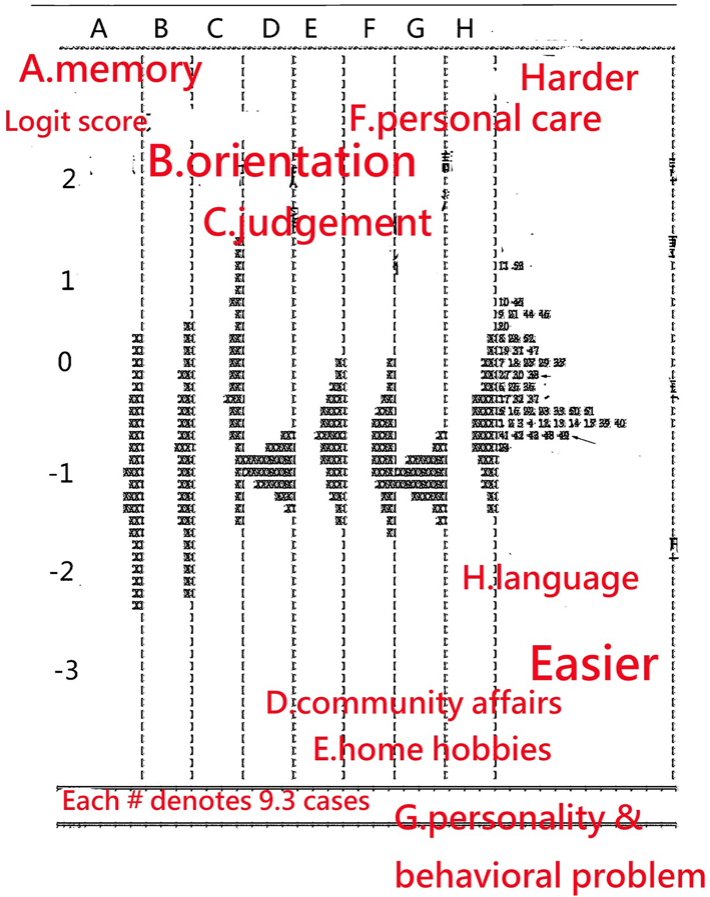
Item and person dispersion on an interval logit continuum scale

The item difficulties are shown on the right-hand side in Fig. 2. The more difficult items (i.e., those with a high logit score, which indicates increased difficulty in providing a response on dementia tendency) are located at the top. The easier items are located at the bottom in Fig. 2. In general, the person logit measures are labeled on the left side on a continuum logit scale in Fig. 2, and the extent of dementia is displayed across subscales in the middle panels in Fig. 2.

All correlation coefficients across subscales are higher than 0.8 (Table 3). The MCAT reliabilities (i.e., Cronbach’s *α* values) across subscales are 0.78, 0.75, 0.73, 0.68, 0.78, 0.75, 0.69, and 0.63, which are higher than those obtained using the separated unidimensional CAT. All MCAT reliabilities are lower than 0.90, indicating that the person–dementia number can be separated into at least three strata.

**Table 3:** Correlation coefficients, reliabilities, variances, and covariances for the eight domains

Domain	A	B	C	D	E	F	G	H
A		0.55	0.55	0.11	0.28	0.29	0.1	0.27
B	0.91		0.64	0.13	0.31	0.32	0.11	0.31
C	0.92	0.94		0.12	0.29	0.29	0.1	0.32
D	0.88	0.92	0.83		0.06	0.07	0.02	0.06
E	0.95	0.93	0.89	0.89		0.16	0.06	0.16
F	0.93	0.91	0.84	0.89	0.96		0.06	0.17
G	0.82	0.8	0.72	0.79	0.86	0.86		0.06
H	0.8	0.79	0.84	0.74	0.86	0.85	0.79	
Variance	0.53	0.69	0.67	0.03	0.16	0.18	0.03	0.22
Reliability^a^	0.78	0.75	0.73	0.68	0.78	0.75	0.69	0.63
Item length	14	6	3	5	5	5	9	6
Reliability^b^	0.72	0.5	0.41	0.43	0.43	0.43	0.69	0.5
Mean in logit	− 0.92	− 0.69	− 0.01	− 0.86	− 0.61	− 0.71	− 0.97	− 0.44
SD	0.02	0.03	0.03	0.01	0.01	0.01	0.01	0.02

Correlation coefficient: lower left; covariance: upper right; ^a^MCAT reliabilities; ^b^CAT reliabilities (i.e., Cronbach’s α values)

### MCAT accuracy and efficiency

**Fig. 3:**
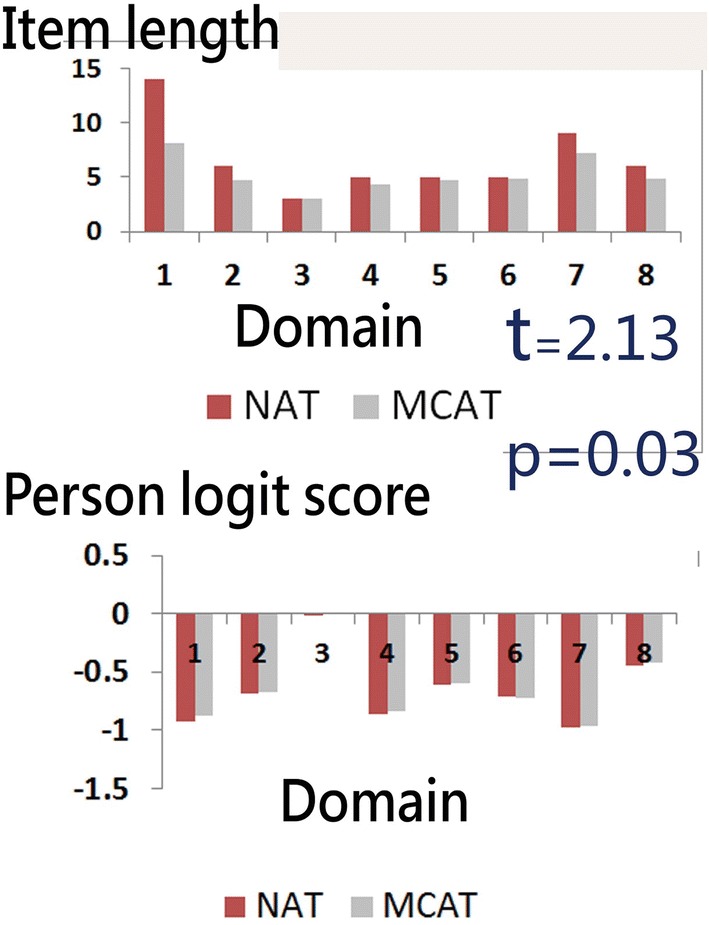
Comparison in efficiency and accuracy among scenarios

The item lengths (i.e., efficiencies) across subscales on NAT and MCAT show a significant difference (*t* = 2.13, *p* < 0.05; Fig. 3). The precision (i.e., the estimated person measures) between NAT and MCAT is equivalent to each other (*p* > 0.05, 2.3%; 9 cases in difference < 18 = 366 × 0.05), thereby indicating that the number of answered items is lower (i.e., more efficient) for MCAT than that for NAT (all 49 items answered) at a rate of approximately 20.19% (= [53 − 42.3]/53) per item length saved. However, the measurement accuracy of MCAT is not compromised. The average number items responded to in MCAT across the eight subscales are 9.2, 4.6, 3.0, 4.6, 4.4, 4.5, 7.2, and 4.8.

### Cut-off points for dementia

**Fig. 4:**
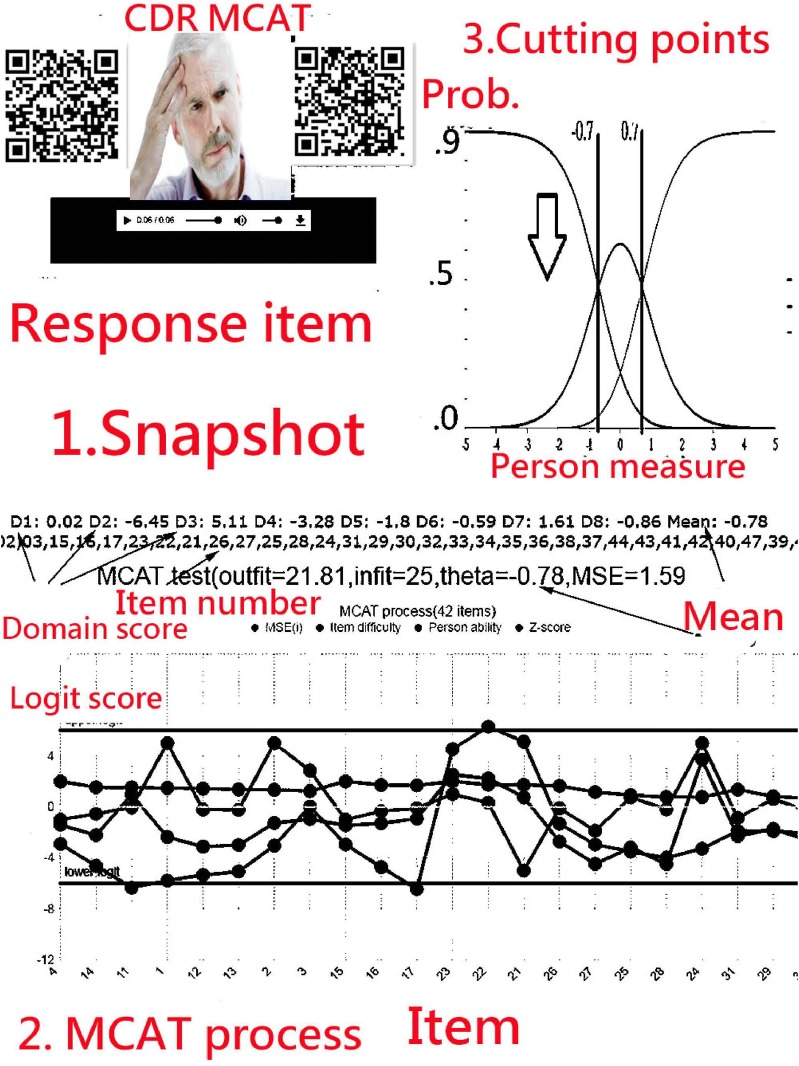
A snapshot of online CDR-MCAT assessment

All persons could be separated into three strata. The cut-off points were set at logits of −0.7 and 0.7. When transforming the probability into a percentile score (i.e., from 0 to 100), the probabilities were located at 0.33 and 0.67, as shown on the top-right bottom in Fig. 4. If five categories are applied (i.e., healthy, very mild, mild, moderate, and severe [13, 14]), the cut-off points can be set at − 1.4, − 0.4, 0.4, and 1.4, respectively.

### Online MCAT assessment

**Fig. 5:**
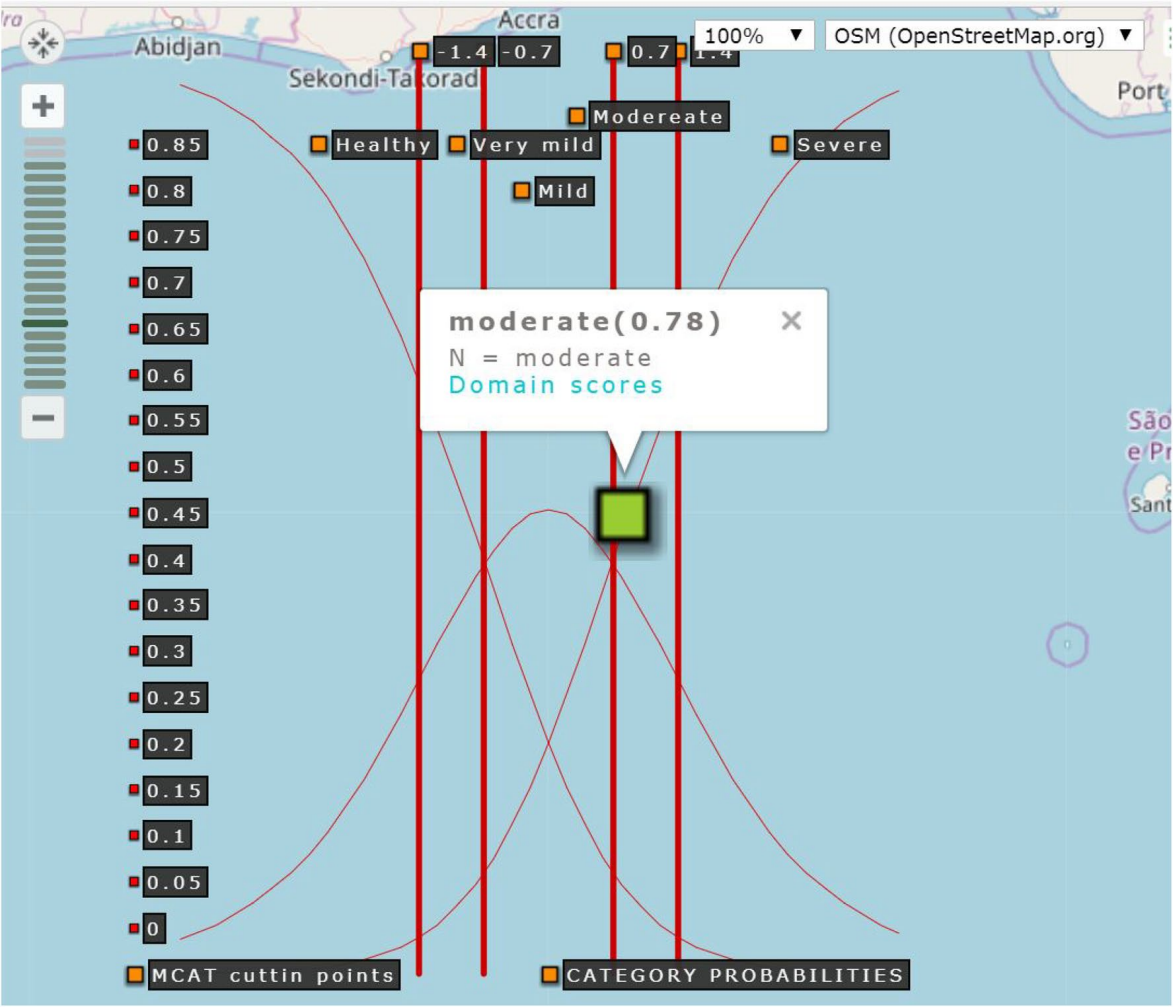
The CDR score shown on the Google maps

After scanning a QR code, the first item was randomly selected, and then, it appeared on a smartphone (Fig. 4). Person domain scores could be estimated using MCAT (Fig. 4). In the MCAT process, the measurement of standard error (MSE) for each subscale decreased when the number of the items increased (Fig. 4). A link to the MCAT video is provided in Additional file 2. Interested readers may scan the QR code in Fig. 4 to perform the CDR-MCAT. The result will open a dashboard on Google Maps (Fig. 5) to allow readers to examine the global score of dementia and the score on each domain.

## Discussion

### Key findings

The results indicate that MCAT is 20.19% (= [53 − 42.3]/53) more efficient for answering questions than NAT. The Cohen effect sizes [30] for MCAT correlation coefficients across subscales are large. The difference number ratio of NAT against MCAT is less than 5% (*p *> 0.05; 2.3%; 9 cases in difference < 18 = 366 × 0.05; paired *t* test), indicating that the precisions (i.e., the estimated person measures) between NAT and MCAT do not differ (i.e., achieving similar precision). The online MCAT dementia APP for patient family members is suitable for smartphones.

### Study contribution

The unstructured CDR questionnaire can be translated into structured subscales, such as those that employ Likert-type scoring [12]. As with all forms of Web-based technology, advances in mobile communication technology are rapidly increasing. However, online MCAT has not been introduced until now even if eight papers were found based on the keywords (multidimensional computerized adaptive testing[Title]) searched in Medline. We verified that online MCAT can be feasible, applicable, and efficient for examining the extent of dementia in health care settings. The results are consistent with computerized assessment using the traditional methods in a previous study [12, 31, 32]. Furthermore, we implemented the IRT algorithm incorporated with MCAT, resulting in a 20.19% enhancement in efficiency and reducing the response time for technicians and family members.

Traditionally, CAT is performed for each subscale separately, whereas subscales are assessed jointly and simultaneously for MCAT. Numerous studies have indicated that unidimensional CAT is more efficient than NAT [7, 8, 31–35]. In general, MCAT is more efficient than separate unidimensional CAT in terms of reducing test length [10, 16, 20]. We confirm that the MCAT-based CDR requires significantly fewer answered items to measure the extent of dementia than NAT without compromising its measurement precision, especially for items with many domains and high correlations among domains [10].

### Implications

The online CDR-MCAT module can be used on personal computers, tablets, or smartphones to predict the extent of dementia in patients. We designated the average logit score among domains as the extent of dementia. These scores correspond to five degrees (healthy, very mild, mild, moderate, and severe [13, 14]) with cut-off points at − 1.4, − 0.4, 0.4, and 1.4, respectively. For each case, an average of 4 min is required to examine the extent of dementia using MCAT, saving 6.5 h for 100 cases in a daily CDR assessment that is performed using NAT. Thus, the online CDR-MCAT is promising for use in clinical practice in the future.

### Strengths of this study

Three goals were achieved in this study: (1) MCAT was verified as 20.19% (= [53 − 42.3]/53) more efficient for answering questions and achieved similar precision in measurements to that of NAT; (2) a set of cut-off points was determined at − 1.4, − 0.4, 0.4, and 1.4, which can be used for reporting the extent of dementia for patients through the process of MCAT; (3) the online MCAT dementia APP for patients’ family members is confirmed to be suitable for smartphones; and the results are directly shown on Google Maps (Fig. 5), a feature that is rarely seen in previously published papers.

In addition, the online animated MCAT APP can be accessed by scanning the QR code in Fig. 4. All detailed information and operation processes regarding this study are provided in Additional file 4.

Furthermore, cut-off points at logits of − 1.4, − 0.4, 0.4, and 1.4 with an equal stratum member size might be generalized to other incidences or diseases when the patient’s true- and false-positive disease-specific status is not known beforehand. Similar to the CDR, we merely intend to identify the grade of the incidence and compare it with the norm.

### Limitations of the study

Several topics should be considered more thoroughly in further research. First, we investigated only the 53-item CDR questionnaire for family members. The 41-item patient version of CDR-MCAT was not presented in this study. We suggest that interested readers view the link in Fig. 4 to manipulate the CDR-MCAT on their own.

Second, the IRT-based Rasch analysis included technical terms such as item difficulty, multidimensional MCAT, Rasch logit score, MSE, and *Z*-score (= [observed score − expected score]/SD), which were not fully explained in this paper due to space limitations.

Third, the number of cut-off points is not limited to three or five strata if the separation index (i.e., Cronbach’s *α*) reaches a sufficiently high level, which affects the determination of appropriate cut-off points for the CDR. The sample is normally distributed with an equal size; thus, we followed the established CDR [13, 14], which includes five degrees (i.e., healthy, very mild, mild, moderate, and severe) to distinguish the degree of dementia.

Fourth, the online MCAT module for CDR implemented in this study has not been completely verified as a wholly perfect process. It should be further modified and streamlined for use in clinical practice and to provide more contributions to researchers and practitioners in the future.

## Conclusion

The online CDR-MCAT can reduce the burden on respondents without compromising measurement precision, and it increases endorsement efficiency. The developed MCAT module is recommended for assessing dementia using the cut-off points for the average domain scores at − 1.4, − 0.4, 0.4, and 1.4 logits to classify the extent of dementia as healthy, very mild, mild, moderate, or severe, respectively.

## Additional files

**Additional file 1.** MP4: Simulation for comparing efficiency and precision between NAT and MCAT. http://www.healthup.org.tw/marketing/course/marketing/simulation_mcat.mp4.

**Additional file 2.** MP4: Online CDR MCAT. http://www.healthup.org.tw/marketing/course/marketing/CDR_MCAT2018.mp4.

**Additional file 3.** MP4: Using Conquest to plot the item-person map. http://www.healthup.org.tw/marketing/course/marketing/CDR_conquest.mp4.

**Additional file 4.** MP4: Briefing on this study. http://www.healthup.org.tw/marketing/course/marketing/CDR_MCAT_proms2017.mp4.

## Data Availability

This research is based on a simulation study. All codes and data can be obtained from those in additional supporting files of this study.

## Abbreviations

APP: application
CASI: cognitive abilities’ screening instrument
CAT: computer adaptive testing
CDR: Clinical Dementia Rating
CTT: classic test theory
DMR: dementia questionnaire for mentally retarded persons
DSDS: dementia scale for Down syndrome
DSQIID: dementia screening questionnaire for individuals with intellectual disabilities
IRT: item response theory
MLE: maximum-likelihood estimation
MMSE: mini-mental state examination
MNSQ: mean-square
MSE: mean-squared error
NAT: non-adaptive testing
PCA: principal component analysis
PTME: point-biserial correlation coefficients on measures
SD: standard deviation
SE: standard error
SEM: standard error measurement
